# Platelet transfusion response in critically ill patients with thrombocytopenia: a retrospective study and predictive nomogram in a general ICU population

**DOI:** 10.1080/07853890.2025.2525395

**Published:** 2025-07-01

**Authors:** Hanyu Ge, Yanqing Liu, Tongyu Li, Rui Lv, Jieyi Wang, Wei You, Danni Song, Shilin Hu, Feng Zhao, Heng Fan, Dingfeng Lv

**Affiliations:** a Department of Blood Transfusion, The First Affiliated Hospital of Ningbo University, Ningbo, Zhejiang, China; b Department of Laboratory Medicine, The First Affiliated Hospital of Ningbo University, Ningbo, Zhejiang, China; c Department of Hematology, The First Affiliated Hospital of Ningbo University, Ningbo, Zhejiang, China; d Department of Intensive Care Unit, The First Affiliated Hospital of Ningbo University, Ningbo, Zhejiang, China; e Department of Blood Transfusion, The Second Affiliated Hospital and Yuying Children’s Hospital of Wenzhou Medical University, Wenzhou, Zhejiang, China

**Keywords:** Platelet transfusion, intensive care units, linear models, nomograms, survival analysis

## Abstract

**Background:**

Although suboptimal platelet transfusion (PT) response in critically ill patients with thrombocytopenia remains a challenge in clinical practice. This study aimed to investigate PT response during intensive care unit (ICU) stay among thrombocytopenic patients without underlying hematologic disease.

**Methods:**

This retrospective single-center analysis included thrombocytopenic patients without primary hematologic disorders who received PT in ICU between June 2021 and December 2023. Clinical and laboratory variables were analyzed using a generalized linear mixed-effects model (GLMM), with the results visualized through a nomogram. The 28-day survival curves, stratified by receiving single or multiple PT episodes, were established using the Kaplan–Meier method.

**Results:**

Suboptimal PT response was observed in 522 episodes (77.9%, 522/670) and in 291 patients (79.9%, 291/364). The GLMM identified sepsis, splenomegaly, mechanical ventilation, higher APACHE II score, and longer time interval of post-PT platelet count as independent predictors of suboptimal response, while higher white blood cell count at ICU admission and the PT episode number in ICU were independently protective. A nomogram based on these seven variables demonstrated good predictive performance. Suboptimal PT episodes were associated with higher red blood cell and fresh frozen plasma requirements. The 28-day survival probability was significantly higher in the single transfusion group with optimal response versus the suboptimal response.

**Conclusions:**

Repeat PT may enhance the PT response and survival. Suboptimal PT response was associated with increased RBC and FFP transfusion requirements. The established nomogram demonstrated strong predictive accuracy and may provide a practical tool for optimizing PT practices in the ICU.

## 1. Introduction

Thrombocytopenia, defined as a platelet count below 150 × 10^9^/L, is a common complication among critically ill patients [1,2]. The prevalence of thrombocytopenia at ICU admission ranges from 8.3% to 67.6%, while its cumulative incidence during ICU stay varies from 13.0% to 44.1% [2]. Recent studies suggest that thrombocytopenia may negatively affect both short- and long-term outcomes in critically ill patients, including increased bleeding, prolonged ICU and hospital stays, and higher mortality [3,4].

Platelet transfusion (PT) is commonly administered to thrombocytopenic patients to increase platelet levels, prevent or mitigate bleeding risk and improve clinical prognosis [3,5]. However, suboptimal PT response, defined as a body surface area (BSA)-adjusted corrected count increment (CCI) below 2.5, occurs commonly in critically ill patients [6–8]. A 9-year retrospective study, which included patients with hematologic malignancies and solid tumors, found that 55.8% (820/1470) of total PT episodes resulted in a poor response [9]. In addition, a prospective multi-central observational study reported suboptimal PT response in 73.9% (349/472) of prophylactic PT episodes and 77.9% (141/181) of patients [7]. In a multicenter randomized trial, Baarle et al. reported catheter-related bleeding (World Health Organization (WHO) grade 2–4) in 9 of 188 thrombocytopenic patients (4.8%) despite PT, with grade 3 or 4 bleeding occurring in 4 patients (2.1%) [10]. Although alloimmunization to human leukocytes antigens (HLA) or platelet specific antigens is a well-established mechanism of platelet transfusion refractoriness (PTR) that can be mitigated through targeted platelet selection strategies, it accounts for less than 10% of PTR cases [11]. In patients with both clinical and alloimmune factors for PTR, the use of HLA-selected platelet components may not substantially improve PT response [12]. Previous studies have highlighted the role of clinical factors in suboptimal PT response in ICU patients. However, these studies have limitations, such as focusing on patients with hematological diseases or tumors [9], failing to adjust for platelet dosage and BSA [6], neglecting the random effects of patient between single and multiple PT episodes [3], and lacking a predictive model or visual tools applicable in clinical practice [7].

Although suboptimal platelet transfusion (PT) response remains a clinical challenge in critically ill patients with thrombocytopenia, limited research has focused on patients without primary hematologic diseases. This study investigated PT response during ICU stay among thrombocytopenic patients without underlying hematologic conditions. Using a retrospective single-center observational study, we seek to evaluate the following objectives: (1) to describe PT response at 10–24 h post-transfusion and identify factors associated with suboptimal PT response, defined as a BSA-adjusted corrected count increment (CCI) below 2.5 [13,14]; (2) to investigate the association between suboptimal PT response and clinical outcomes, including mortality and bleeding events; and (3) to construct and evaluate the clinical utility of a nomogram for predicting and visualizing suboptimal PT response.

## 2. Methods

### 2.1. Study design and population

We conducted a retrospective single-center study in six tertiary medical ICUs at the First Affiliated Hospital of Ningbo University from June 2021 to December 2023. The hospital, spread across three campuses in Ningbo City, comprises three medical ICUs, two surgical ICUs, and one general ICU, with a bed capacity ranging from 16 to 37 beds per unit.

**Figure 1.**
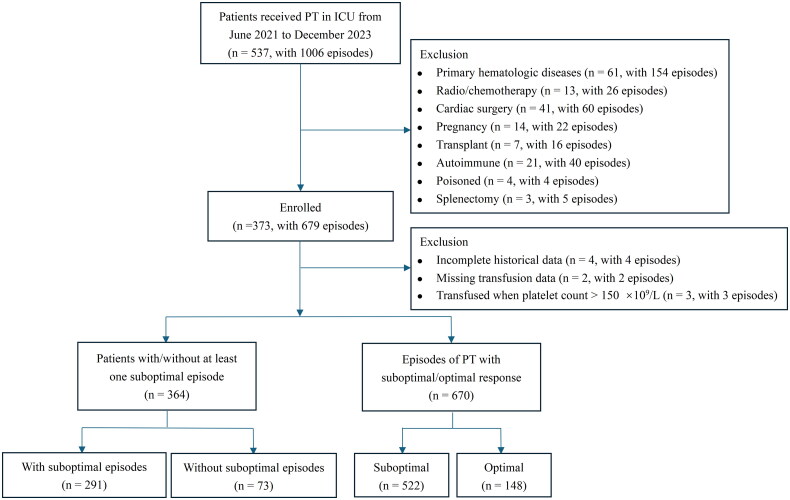
Inclusion flowchart of patients and platelet transfusion episodes. PT platelet transfusion, ICU intensive care unit.

Patients over 14 years of age with thrombocytopenia, as defined by a platelet count below 150 × 10^9^/L, who received PT during their ICU stay were included. PT were given in accordance with the clinical transfusion guidelines and physician judgment. The primary indications included: (1) prophylactic transfusion for non-bleeding patients with platelet counts <10 × 10^9^/L; (2) platelet counts <50 × 10^9^/L in patients undergoing invasive procedures or with active bleeding; and (3) platelet counts <100 × 10^9^/L in cases of major trauma or neurosurgical conditions. The exclusion criteria were: (1) Patients with primary hematologic diseases; (2) Patients who received radiotherapy and/or chemotherapy within the past six months; (3) Patients who underwent cardiac surgery; (4) Patients with pregnancy; (5) Patients who underwent organ transplant, or stem cell transplantation; (6) Patients with active autoimmune disease; (7) Patients with food and/or drug poisoning; (8) Patients who underwent splenectomy; (9) Patients with incomplete historical data. Exclusions were all limited to the hospitalization period. All episodes of PT were ABO-compatible, and no additional HLA-matched transfusions were included. Multiple consecutive PT episodes administered within the interval between two complete blood count tests and less than 8 h apart, were considered a single episode, with the platelet doses aggregated. The inclusion flowchart of patients and PT episodes is illustrated in Figure 1.

The study was conducted according to the guidelines of the Helsinki Declaration of 2013. Due to the retrospective nature of the study, the Ethics Committee of the First Affiliated Hospital of Ningbo University waived the need of obtaining informed consent. Additionally, the study was registered in the National Medical Research Registration and Filing Information System (MR-33-24-027153).

### 2.2. Data collection

Patient characteristics were extracted from the medical records and medical data center of the hospital. We distinguished between static characteristics present upon ICU admission and dynamic features that were likely to evolve during the ICU stay. Static characteristics included demographic factors such as age, gender, body mass index, BSA, ABO blood group and ICU admission diagnoses (hypertension, diabetes mellitus, heart failure, liver failure, renal failure, respiratory failure, splenomegaly (a spleen length >12 cm, a thickness >4 cm, or a volume >400 cm³), solid tumors, sepsis, shock, and trauma with brain injury) were recorded. Patients’ ICU admissions were categorized into medical, scheduled surgical and unscheduled surgical. The severity of illness and organ failures at ICU admission were assessed using the Glasgow Coma Scale (GCS) [15], the Acute Physiology and Chronic Health Evaluation II (APACHE II), the Simplified Acute Physiology Score II (SAPS II) [16] and the Sequential Organ Failure Assessment (SOFA) [17]. Platelet count, hemoglobin level, prothrombin time and international normalized ratio (INR) at ICU admission were also collected. Dynamic data were related to the clinical course during the ICU stay and especially to the concurrent clinical status in every episode of PT, including biological parameters, clinical and laboratory evaluations, medicine administrations and features of transfusion episode.

Data related to platelet concentrates were retrieved from the Department of Blood Transfusion of the First Affiliated Hospital at the Ningbo University. These data included the duration of platelet storage, the platelet dose (dosage of platelets administered), and ABO compatibility of the platelets.

### 2.3. Donor platelet collection and storage parameters

Only ABO-compatible apheresis platelets were administered to patients. These platelets were collected from healthy blood donors, aged 18 to 55, at the Ningbo Central Blood Station in Zhejiang Province, China. None of the donors had taken aspirin or other medications affecting platelet function for two weeks prior to donation. The platelet counts of all donors ranged from 250 to 450 × 10^9^/L, meeting the criteria for double platelet collection. Each unit contained a minimum of 2.5 × 10^11^ platelets and fewer than 1.0 × 10^6^ leukocytes. The volume of each apheresis platelet unit varied between 200 and 400 ml. The platelet concentrates were stored at temperatures between 20 °C and 24 °C, with continuous slow agitation. The shelf life of the platelet concentrates was 7 days from the date and time of collection.

### 2.4. PT response and outcomes

PT response was evaluated using the CCI, calculated as follows:

CCI = [(post-transfusion platelet count) – (pre-transfusion platelet count)] × BSA/(dosage of platelets transfused). A CCI of less than 2.5 at 10–24 h post-transfusion was defined as a suboptimal PT response [8,13].

Patients were deemed PTR if two consecutive ABO-compatible transfusions resulted in a suboptimal PT response.

Clinical outcomes included ICU mortality, 28-day survival, and the length of stay in both the ICU and hospital. Bleeding events were recorded according to the WHO classification for grades 3–4. Data on blood component requirements, including the total times and units of PT during the ICU stay, as well as red blood cells (RBCs) and fresh frozen plasma (FFP), were collected as indicators of major bleeding following PT.

### 2.5. Statistical analysis

Continuous variables were presented as median and interquartile ranges (25th–75th percentile) and categorical variables as number and percentage (n, %). The characteristics of patients with suboptimal PT response were compared to those without suboptimal PT response. Characteristics of episodes of PT with and without suboptimal platelet increment were also compared. The Mann–Whitney U test was used for quantitative and ordinal categorical data, and either the Chi-square or Fisher’s exact test was applied for qualitative data, as appropriate. Univariate and multivariate generalized linear mixed-effect model (GLMM) were used to account for repeated factors within individual patients and assess the association between patient and episode characteristics and PT response. The GLMM was constructed with a logit link, binary distribution, a random effect for patients to account for repeated transfusions, and fixed effects for other variables. Variables incorporated into the multivariate GLMM were sequentially selected based on LASSO regression, stepwise regression, and their potential impact on PT efficacy. Odds ratios (ORs) with 95% confidence intervals were reported. Variables exhibiting high collinearity were primarily eliminated through correlation matrix analysis (absolute correlation coefficient >0.7) and by excluding those with a variance inflation factor (VIF) greater than 5. Continuous variables were standardized, and categorical variables were converted into dummy variables prior to performing LASSO regression and stepwise regression. Due to the standardization of continuous variables, the resulting forest plot may have limited clinical applicability, so a nomogram was constructed using the original data for the selected variables, using the rms package in R. The receiver operating characteristic (ROC) curve, the calibration curve and decision curve analysis (DCA) were also performed. The 28-day survival curves were stratified based on single and multiple episodes of PT. Patients receiving a single transfusion were categorized into effective (CCI ≥ 2.5) and ineffective (CCI <2.5) groups according to their CCI outcomes, with the observation period commencing from the time of transfusion. And patients receiving multiple transfusions were divided into PTR and non-PTR groups. Observation for PTR patients began after the second consecutive suboptimal transfusion, while for non-PTR patients, it began after the second transfusion.

To enhance generalizability and robustness of the model, a sensitivity analysis was conducted by comparing models with and without ward-level as a second random effect.

All statistical analyses were performed using R (version 4.3.2; http://www.r-project.org) with the following packages: lme4, Matrix, polspline, multcomp, survminer, rms, dplyr, glmnet, Hmisc, car, caret, broom.mixed, and rmda. Additionally, SPSS version 27.0 (SPSS Inc., Chicago, IL, USA) was used. Two-sided tests were performed, and statistical significance was considered at *p* value <0.05.

## 3. Results

### 3.1. Patient characteristics

A total of 537 patients who received PT in the ICU at the First Affiliated Hospital of Ningbo University between June 2021 and December 2023 underwent 1006 episodes of PT. Of these, 364 patients with 670 episodes were included in the analysis according to the inclusion and exclusion criteria (Figure 1).

Baseline characteristics of the 364 patients were displayed in Table 1. They were mainly male (67.5%) with a median age of 67 (53–77) years and a median BSA of 1.79 m^2^ (1.66–1.90). Overall, 163 patients (44.7%) were admitted under the medical category and accounted for the majority of PT episodes (42.2%, 283/670). Respiratory failure was the most common primary ICU admission diagnosis, affecting 290 patients (79.6%). The median GCS score was 3 (3–5), while the median APACHE II score was 22 (18–27), the median SAPS II score was 59 (46–70), and the median SOFA score was 11 (9–13). Upon ICU admission, the median platelet count was 75.50 × 10^9^/L (42.25 × 10^9^–123 × 10^9^), and the median hemoglobin level was 96.5 g/L (76–117).

**Table 1.** Baseline characteristics of patients with and without suboptimal platelet transfusion response.

Variables	With suboptimal response to PT *n* = 291	Without suboptimal response to PT *n* = 73	Test value (*Z*/χ^2^)	*p* value
Demographic characteristics				
Age, years	67.0 (53–76)	66.0 (52.5–78.5)	−0.089	0.929
Male sex	200 (68.7%)	46 (63.0%)	0.870	0.351
Body surface area, m^2^	1.79 (1.66–1.90)	1.77 (1.65–1.90)	0.567	0.570
Diagnosis on ICU admission				
Liver failure	191 (65.6%)	38 (52.1%)	4.613	0.032
Renal failure	198 (68.0%)	40 (54.8%)	4.525	0.033
Respiratory failure	235 (80.8%)	55 (75.3%)	1.056	0.304
Splenomegaly	30 (10.3%)	2 (2.7%)	4.170	0.041
Sepsis	158 (84.5%)	29 (15.5%)	4.959	0.026
Admission category			6.688	0.035
Medical	130 (44.7%)	33 (45.2%)		
Scheduled surgical	64 (22.0%)	25 (34.2%)		
Unscheduled surgical	97 (33.3%)	15 (20.5%)		
Patient ABO Group				
A	83 (28.5%)	27 (37.0%)	2.603	0.457
B	76 (26.1%)	15 (20.5%)		
O	104 (35.7%)	26 (35.6%)		
AB	28 (9.6%)	5 (6.8%)		
Clinical and laboratory evaluations on ICU admission				
APACHE II score, points	24.0 (19.0–28.0)	16.0 (11.5–21.0)	7.863	<0.001
SAPS II score, points	60.0 (48.0–72.0)	52.0 (40.0–60.5)	3.965	<0.001
SOFA score, points	11 (9–13)	10 (8–11)	3.561	<0.001
CRRT	64 (22.0%)	8 (11.0%)	4.478	0.034
Platelet count, × 10^9^/L ref: 150–400	77.0 (44.0–126.0)	80.0 (45.0–125.5)	−0.294	0.769
Hemoglobin, g/L ref: 130–175	97.0 (77.0–118.0)	101.0 (78.5–119.0)	−0.852	0.394
WBC count, ×10^9^/L	11.1 (7.3–16.5)	11.5 (7.56–18.7)	−0.697	0.486
Thrombin time > 16.6 s ref: 10.3–16.6	214 (73.5%)	45 (61.6%)	4.024	0.045
INR > 1.2 ref: 0.90–1.20	196 (67.4%)	40 (54.8%)	4.038	0.044
Clinical outcomes				
Platelet units transfused, U	10 (10–20)	10 (10–10)	5.225	<0.001
Total times of PT in ICU	1 (1–2)	1 (1–1)	5.254	<0.001
ICU length of stay, days	10.6 (5.2–21.6)	11.3 (5.7–23.6)	−0.098	0.922
Hospital length of stay, days	18.2 (9.0–35.7)	21.8 (11.7–33.3)	−0.487	0.626
ICU mortality	190 (65.3%)	42 (57.5%)	1.520	0.218

PT platelet transfusion, APACHE II Acute Physiology and Chronic Health Evaluation II, SAPS II Simplified Acute Physiology Score II, SOFA Sequential Organ Failure Assessment, CRRT continuous renal replacement therapy, ref reference value, WBC white blood cell, INR international normalized ratio.

### 3.2. Patient‑related characteristics and suboptimal PT response

Patients with suboptimal PT response were more likely to have liver failure (65.6% versus (vs.) 52.1%, *p* = 0.032), renal failure (68.0% vs. 54.8%, *p* = 0.033), and splenomegaly (10.3% vs. 2.7%, *p* = 0.041). They also had a higher incidence of sepsis (84.5% vs. 15.5%, *p* = 0.026). In terms of clinical severity, patients with suboptimal response had significantly higher APACHE II score (24.0 (19.0–28.0) vs. 16.0 (11.5–21.0), *p* < 0.001), SAPS II (60.0 (48.0–72.0) vs. 52.0 (40.0–60.5), *p* < 0.001), and SOFA score (11 (9–13) vs. 10 (8–11), *p* < 0.001). Additionally, these patients were more likely to undergo continuous renal replacement therapy (CRRT) (22.0% vs. 11.0%, *p* = 0.034) and exhibited prolonged thrombin time (>16.6 s (s)) (73.5% vs. 61.6%, *p* = 0.045) and elevated INR (>1.2) (67.4% vs. 54.8%, *p* = 0.044) at ICU admission. PT requirements were higher in the suboptimal response group (10 units (10–20) vs. 10 units (10–10), *p* < 0.001), as was the total times of PT during ICU stay (1 time (1–2) vs. 1 time (1–1), *p* < 0.001), and they received larger volumes of FFP (1010 mL (410–1920) vs. 680 mL (150–1470), *p* = 0.015) (Table 1).

### 3.3. Episode‑related features and suboptimal PT response

Episodes of PT with suboptimal response were associated with significantly lower mean arterial pressure (81.3 mmHg (73.3–91.7) vs. 85.2 mmHg (75.1–95.3), *p* = 0.035), reduced 24-hour urine output (1440.0 mL (157.5**–**2600.0) vs. 1725.0 mL (812.5–2750.0), *p* = 0.038), and a higher incidence of CRRT (28.5% vs. 17.6%, *p* = 0.007). Patients in these episodes also exhibited higher severity scores, including APACHE II score (22 (19–27) vs. 17 (12–21), *p* < 0.001), SAPS II (59 (48–69) vs. 56 (44–65), *p* = 0.017), and SOFA score (11 (9–13) vs. 10 (9–12), *p* = 0.024) on application of PT. Laboratory values before transfusion indicated lower potential of hydrogen (7.42 (7.37–7.47) vs. 7.44 (7.40–7.48), *p* = 0.043), bicarbonate levels (22.5 mmol/L (18.6–26.3) vs. 23.5 mmol/L (20.0–27.9), *p* = 0.027), calcium (1.09 mmol/L (1.02–1.17) vs. 1.13 mmol/L (1.05–1.19), *p* = 0.010), base excess (–1.8 mmol/L (–5.8–2.53) vs. 0.15 mmol/L (–3.4–4.1), *p* < 0.001) and platelet counts (24 × 10^9^/L (15.0 × 10^9^–38.0 × 10^9^) vs. 30.5 × 10^9^/L (20.0 × 10^9^–49.0 × 10^9^), *p* < 0.001), alongside higher lactate levels (2.2 mmol/L (1.4–4.0) vs. 1.9 mmol/L (1.3–3.2), *p* = 0.045) and anion gap (11 mmol/L (7–15) vs. 10 mmol/L (7–14), *p* = 0.049). Additionally, coagulation abnormalities were more frequent in the suboptimal response group, including elevated activated partial thromboplastin time (APTT) (>36.5 s) (45.6% vs. 32.4%, *p* = 0.004), INR (>1.2) (70.3% vs. 60.1%, *p* = 0.019) and thrombin time (>16.6 s) (69.3% vs. 57.4%, *p* = 0.007), as well as reduced fibrinogen levels (<2 g/L) (33.3% vs. 23.6%, *p* = 0.025) (Table 2).

**Table 2.** Comparison of platelet transfusion characteristics with and without suboptimal response defined by a CCI below 2.5.

Variables	Suboptimal PT response *n* = 522	Optimal PT response *n* = 148	Test value (*Z*/χ^2^)	*p* value
*Biological features before transfusion*				
Heart rate, /min	92 (78–106)	89 (78–106)	0.495	0.620
Temperature, °C	36.7 (36.0–37.3)	36.8 (36.1–37.3)	−0.325	0.745
Mean arterial blood pressure, mmHg	81.3 (73.3–91.7)	85.2 (75.1–95.3)	−2.112	0.035
24 h urine volume, mL	1440.0 (157.5–2600.0)	1725.0 (812.5–2750.0)	−2.076	0.038
Mechanical ventilation	455 (87.2%)	129 (87.2%)	0.000	0.999
CRRT	149 (28.5%)	26 (17.6%)	7.199	0.007
*Clinical and laboratory evaluations before transfusion*				
APACHE II score, points	22 (19–27)	17 (12–21)	9.773	<0.001
SAPS II score, points	59 (48–69)	56 (44–65)	2.388	0.017
SOFA score, points	11 (9–13)	10 (9–12)	2.255	0.024
Potential of hydrogen	7.42 (7.37–7.47)	7.44 (7.40–7.48)	−2.021	0.043
Actual bicarbonate, mmol/L	22.5 (18.6–26.3)	23.5 (20.0–27.9)	−2.214	0.027
Hemoglobin, g/L	77.0 (68.0–90.0)	80.5 (71.0–92.0)	−1.780	0.075
Platelet count, × 10^9^/L	24.0 (15.0–38.0)	30.5 (20.0–49.0)	−3.665	<0.001
White blood cell count, × 10^9^/L	9.2 (5.1–13.7)	9.2 (6.5–14.0)	−0.850	0.395
Neutrophil percentage, %	87.5 (80.9–91.8)	85.0 (79.3–89.4)	2.874	0.004
Lymphocyte percentage, %	6.6 (3.9–11.0)	7.4 (4.8–12.1)	−1.793	0.073
Monocyte percentage, %	4.4 (2.8–6.8)	5.7 (3.8–8.1)	−4.200	<0.001
APTT > 36.5 s	238 (45.6%)	48 (32.4%)	8.164	0.004
Thrombin time > 16.6 s	362 (69.3%)	85 (57.4%)	7.373	0.007
INR > 1.2	367 (70.3%)	89 (60.1%)	5.488	0.019
Fibrinogen <2 g/L	174 (33.3%)	35 (23.6%)	5.039	0.025
*Medicine administration*				
Glucocorticoids	147 (28.2%)	50 (33.8%)	1.756	0.185
Polymyxins	57 (10.9%)	7 (4.7%)	5.113	0.024
Procoagulant agents	132 (25.3%)	25 (16.9%)	4.530	0.033
*Features of transfusion episodes*				
PT episode number in ICU	1 (1–3)	1 (1–3)	0.239	0.811
Storage duration of platelet, days	2.8 (2.7–3.7)	2.9 (2.7–3.7)	−1.374	0.169
Time interval of pre-PT count, days	0.29 (0.24–0.36)	0.29 (0.24–0.34)	0.388	0.698
Time interval of post-PT count, days	0.66 (0.51–0.72)	0.66 (0.47–0.72)	0.466	0.641
Length of ICU stay at PT, days	4.0 (2.0–12.3)	4.2 (2.1–8.6)	0.416	0.677
Length of hospital stay at PT, days	5.1 (2.8–16.2)	5.8 (3.1–13.2)	−0.164	0.870

PT platelet transfusion, CRRT continuous renal replacement therapy, APACHE II Acute Physiology and Chronic Health Evaluation II, SAPS II Simplified Acute Physiology Score II, SOFA Sequential Organ Failure Assessment, APTT activated partial thromboplastin time, INR international normalized ratio, ICU intensive care unit.

### 3.4. Factors independently associated with suboptimal PT response

In the univariate analysis, several factors were significantly associated with suboptimal PT response, including diabetes mellitus (OR: 2.81 [1.42–5.54], *p* = 0.003), liver failure (OR: 1.74 [1.03–2.91], *p* = 0.037) and sepsis (OR: 6.44 [3.09–13.43], *p* < 0.001). Higher APACHE II score (OR: 1.19 [1.13–1.25], *p* < 0.001) and prolonged APTT (>36.5 s) (OR: 1.99 [1.23–3.22], *p* = 0.005) were also linked to poorer transfusion response. Additionally, polymyxin administration (OR: 2.71 [1.02–7.18], *p* = 0.045) was also identified as an additional significant predictor (Table 3).

**Table 3.** Univariate and multivariate generalized linear mixed-effect model to evaluate association between demographic and transfusion-related characteristics with suboptimal response.

	Univariate analysis			Multivariate analysis		
Variables	OR	95%CI	*p* value	OR	95%CI	*p* value
*Baseline characteristics*						
*Demographic characteristics*						
* *Age, years	1.00	[0.99–1.02]	0.709			
* *Male sex	0.59	[0.34–1.02]	0.061			
* *Body surface area, m^2^	1.56	[0.40–6.03]	0.519			
* *Diabetes mellitus	2.81	[1.42–5.54]	0.003	1.52	[0.64–3.58]	0.343
* *Liver failure	1.74	[1.03–2.91]	0.037	1.10	[0.55–2.18]	0.795
* *Splenomegaly	3.45	[0.99–11.98]	0.052	5.71	[1.13–28.97]	0.035
* *Sepsis	6.44	[3.09–13.43]	<0.001	4.36	[1.85–10.26]	0.001
* *Admission category (ref = ‘medical’)	1.00	[0.75–1.32]	0.982			
*Clinical and laboratory evaluations on ICU admission*						
* *APACHE II score, points	1.19	[1.13–1.25]	<0.001	3.72	[2.20–6.29]	<0.001
* *WBC count, × 10^9^/L	0.97	[0.94–1.00]	0.025	0.69	[0.50–0.95]	0.023
*Transfusion-related characteristics*						
*Biological features before transfusion*						
* *Mean arterial blood pressure, mmHg	0.98	[0.96–1.00]	0.014	0.87	[0.64–1.18]	0.380
* *CRRT	1.90	[1.06–3.38]	0.030	1.36	[0.63–2.95]	0.439
* *Mechanical Ventilation	1.06	[0.52– 2.15]	0.873	3.71	[1.29–10.70]	0.015
*Transfusion episode-related features*						
SAPS II score, points	1.02	[1.00–1.03]	0.036			
Calcium, mmol/L	0.15	[0.03–0.66]	0.012	0.97	[0.70–1.35]	0.867
Hemoglobin, g/L	0.99	[0.98–1.01]	0.306	1.10	[0.78–1.55]	0.577
Platelet count, × 10^9^/L	0.99	[0.98–1.00]	0.016	0.99	[0.72–1.34]	0.927
Neutrophil percentage, %	1.03	[1.01–1.05]	0.013	1.26	[0.90–1.76]	0.174
Monocyte percentage, %	0.86	[0.80–0.93]	<0.001	0.80	[0.57–1.14]	0.223
APTT > 36.5s	1.99	[1.23–3.22]	0.005	0.99	[0.73–1.35]	0.968
*Medicine administration*						
Glucocorticoids	0.69	[0.41– 1.14]	0.149	0.57	[0.30–1.08]	0.086
Polymyxins	2.71	[1.02–7.18]	0.045	1.43	[0.43–4.78]	0.558
Procoagulant agents	1.89	[1.04–3.42]	0.036	1.60	[0.75–3.38]	0.222
*Features of transfusion episodes*						
PT episode number in ICU	0.96	[0.85– 1.09]	0.550	0.53	[0.32–0.88]	0.014
Time interval of post-PT count, hours	1.59	[0.62– 4.07]	0.334	1.40	[1.01–1.93]	0.041

ICU intensive care unit, APACHE II Acute Physiology and Chronic Health Evaluation II, WBC white blood cell, CRRT continuous renal replacement therapy, SAPS II Simplified Acute Physiology Score II, APTT activated partial thromboplastin time, PT platelet transfusion.

**Figure 2.**
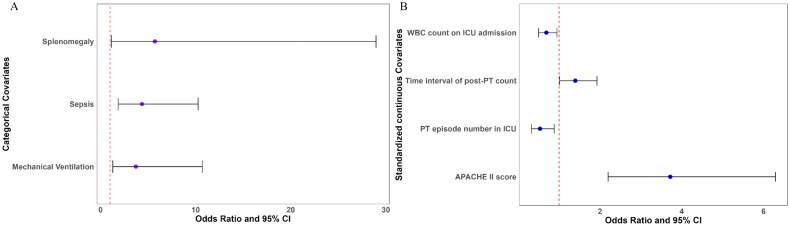
Forest plots evaluate the association between episode-related characteristics with platelet transfusion response. **A.** Forest plot for categorical covariates; **B.** Forest plot for standardized continuous covariates. WBC white blood cell, ICU intensive care unit, PT platelet transfusion.

Variables exhibiting high collinearity were primarily eliminated through correlation matrix analysis (with an absolute correlation coefficient >0.7) (Additional file 1: Figure S1) and by excluding those with a VIF greater than 5 (Additional file 1: File S1). Continuous variables were standardized, and categorical variables were converted into dummy variables prior to performing LASSO regression (Additional file 1: Figure S2A) and stepwise regression (Additional file 1: Figure S2B) to select the variables incorporated into the multivariate GLMM. Subsequently, in the multivariate GLMM analysis, sepsis (OR: 4.36 [1.85–10.26], *p* = 0.001), splenomegaly (OR: 5.71 [1.13–28.97], *p* = 0.035), mechanical ventilation (OR: 3.71 [1.29–10.70], *p* = 0.015), higher APACHE II score (OR: 3.72 [2.20–6.29], *p* < 0.001), and longer time interval of post-PT platelet count (OR: 1.40 [1.01–1.93], *p* = 0.041) were identified as independent risk factors for suboptimal PT response, while higher white blood cell (WBC) count at ICU admission (OR: 0.69 [0.50–0.95], *p* = 0.023) and the PT episode number in ICU (OR: 0.53 [0.32–0.88], *p* = 0.014) were independently protective (Table 3; Figure 2).

### 3.5. Establishment of the nomogram prediction model

**Figure 3.**
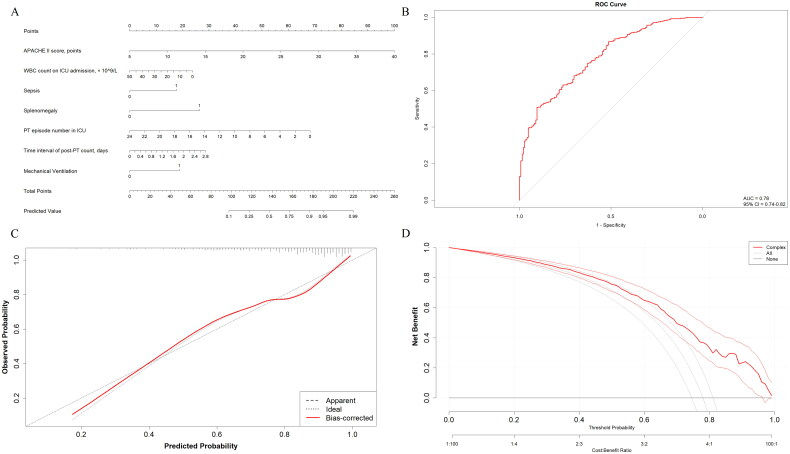
Development of the predictive nomogram. **A.** Predictive nomogram for estimating the response of the platelet transfusion. **B.** Receiver operating characteristic curve of the nomogram. **C.** Calibration curve of the nomogram. **D.** Decision curve analysis of the nomogram.

A nomogram based on the above seven variables was developed and presented in Figure 3A. The optimal cutoff value for the total scores was calculated using ROC. When the cutoff value was 115 points, this scoring system showed a good predictive performance in distinguishing suboptimal PT response from optimal PT response with an AUC of 0.781 [0.741–0.821] (Figure 3B). The corresponding specificity and sensitivity were 79.6% (76.6–83.4) and 70.5% (68.2–75.3), respectively. The calibration curve demonstrated that the predicted line overlapped well with the reference line, indicating a good performance of the predictive nomogram in this patient population (Figure 3C). In addition, the DCA was applied to evaluate the net benefit of the predictive nomogram to verify the clinically utility of the model. Results showed that patients would benefit more over the ‘treat-all’ or ‘treat-none’ strategy when the threshold probability was exceeded 0.8 (Figure 3D).

### 3.6. Clinical outcomes and survival by PT response

**Figure 4.**
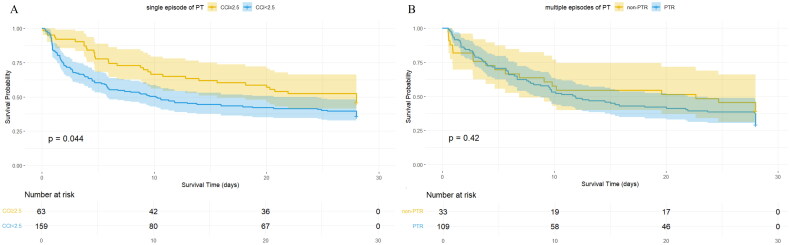
The 28-day survival curves of the patients stratified based on single and multiple platelet transfusions. **A.** Patients received single episode of PT during ICU stay. **B.** Patients received multiple episodes of PT during ICU stay. PT platelet transfusion, CCI corrected count increment, PTR platelet transfusion refractoriness.

No significant differences were observed in ICU or hospital length of stay, or in ICU mortality between the patients with and without suboptimal PT response (Table 1). Whereas, episodes of suboptimal PT response were associated with a higher requirement for RBCs (OR: 1.29 [1.08–1.52], *p* = 0.004) and FFP (OR: 1.00 [1.00–1.00], *p* = 0.009) within the 24 h post-PT (Table 4). Severe bleeding events (WHO grade 3 to 4) occurred at similar rates between two groups (OR: 1.03 [0.61–1.73], *p* = 0.918) (Table 4). In addition, when stratified by whether received a single or multiple PT episodes, the 28-day survival probability of patients was significantly lower in the single transfusion group with a suboptimal response compared to those with optimal response group (log-rank test, *p* = 0.044) (Figure 4A). However, no significant difference in 28-day survival was observed between the PTR and non-PTR groups (log-rank test, *p* = 0.420) (Figure 4B).

**Table 4.** RBC and FFP transfusion requirement and bleeding events within the 24 h after platelet transfusion according to platelet transfusion response.

Variables	Suboptimal PT response *n* = 522	Optimal PT response *n* = 148	Univariate analysis		
			OR	95% CI	*p* value
Requirement of RBC, U	0 (0–2.0)	0 (0–1.5)	1.29	[1.08–1.52]	0.004
Requirement of FFP, ml	0 (0–370)	0 (0–0)	1.00	[1.00–1.00]	0.009
WHO grade 3–4 bleeding	327 (62.6%)	89 (60.1%)	1.03	[0.61–1.73]	0.918

RBCs red blood cells, FFP fresh frozen plasma, PT platelet transfusion.

*p* values comparing episodes are tested by univariate generalized linear mixed-effect model.

### 3.7. Sensitivity analysis

A sensitivity analysis was conducted by comparing two models: one with patient-level random effects and another with both patient- and ward-level random effects. The ‘ward-level’ refers to the six distinct ICU units (medical, surgical, and general ICUs) across three campuses, representing variability in patient populations, clinical protocols, and unit-level practices that may impact PT response. The comparison aimed to assess whether adding ward as a second random effect improved model fit and influenced fixed-effect estimates. The fixed effect estimates were consistent across both models (Additional file 1: File S2 and S3). The comparison of model fit metrics revealed no substantial improvement with the inclusion of ward-level random effects (Akaike Information Criterion, 589 vs. 591; Bayesian Information Criterion, 698 vs. 704), and the likelihood ratio test comparing the two models yielded a *p* value of 0.989 (Additional file 1: Table S1).

## 4. Discussion

In this study, we put insights into PT response in thrombocytopenic patients without primary hematologic disorders during ICU stay, addressing a gap in previous research, which has primarily focused on hematologic thrombocytopenia, where the underlying disease significantly affects PT outcomes [7,9]. Our findings indicated that suboptimal PT response remains a significant challenge in this patient population, with nearly 80% of patients (291/364) experiencing poor platelet increments. A key finding of our study was the identification of several clinical factors associated with suboptimal PT response. Sepsis, splenomegaly, mechanical ventilation, higher APACHE II score, and longer time interval of post-PT platelet count were identified independently associated with suboptimal PT response, while a higher WBC count at ICU admission and the PT episode number in ICU were independently protective. We also explored the clinical outcomes of suboptimal PT response and found no significant differences in ICU or hospital mortality or severe bleeding events (WHO grade 3–4) between patients with and without suboptimal responses. However, episodes of suboptimal PT response had significantly higher requirements for RBC- and FFP transfusions according to the univariate GLMM analysis. Furthermore, a stratified 28-day survival analysis by single and multiple transfusions revealed differing outcomes. In patients received a single transfusion, suboptimal response was linked to reduced survival, whereas in those received multiple transfusions, overall survival was unaffected, even among patients classified as PTR. The sensitivity analysis confirmed that the inclusion of ward as a second random effect did not significantly improve the model’s performance. The GLMM incorporating only patient-level random effects proved to be both robust and generalizable and suitable for use across multiple wards without compromising its accuracy or applicability.

Suboptimal PT responses have been documented in critically ill patients. In a prospective multicentral observational study, Reizine et al. reported a 77.9% rate (141/181) of poor PT response using similar criteria [7]. However, 53.0% patients (96/181) had a hematologic malignancy, and these patients received the majority of episodes of PT in this study (56.8%, 268/472), which limited the generalizability of their findings to general ICU populations. Besides, the suboptimal PT response in their study was defined as CCI <7 at 18–24 h after PT, which was much higher than this in our study. The same definition was applied in a single-center study, Arnold et al. also observed a high rate of poor platelet response but used a shorter time window of 5 h post-transfusion to assess platelet increments [9]. The lack of consensus on the optimal timing for measuring platelet response complicates comparisons across studies. In a review, the suggested CCI cut-offs for defining clinical refractoriness vary by timing, with a CCI of less than 5 at 1 h post-transfusion and less than 2.5 at 16–24 h [8]. In our study, a 10 to 24-h post-transfusion window was chosen, in line with commonly accepted guidelines for evaluating CCI, but this may have contributed to the relatively lower rate of poor responses. Our study conducted in six ICUs, differing in patient population primarily focused on patients without primary hematologic disorders, making our findings more applicable to general ICU populations.

A key contribution of our study was identifying clinical factors influencing PT response in a general ICU population. APACHE II score, an established measure of disease severity in ICU, emerged as the most significant predictor of PT response, indicating that PT response is closely tied to the patient’s overall condition. This finding aligns with previous studies linking SOFA score and SAPS II to poor PT outcomes [7,9]. Though in our analysis, SOFA score and similar metrics were excluded from multivariate GLMM due to collinearity with APACHE II score. The time interval for post-PT platelet counts was identified as a secondary risk factor, reinforcing the importance of timing in CCI measurements, as highlighted above. Splenomegaly was also found to be a significant risk factor, consistent with studies showing the spleen’s role in platelet sequestration, such as Aster’s work using 51Cr-labeled platelets, which demonstrated the spleen as the primary site of platelet accumulation through surface scintillation scanning [18]. Sepsis was also a major factor, driving increased platelet consumption and impairing hemostasis, likely through mechanisms such as disseminated intravascular coagulation and direct platelet–pathogen interactions [19,20]. Additionally, mechanical ventilation was identified as an independent risk factor for suboptimal PT response, consistent with previous research linking low platelet levels to increased mortality in sepsis-associated acute respiratory failure [21]. Interestingly, we found that the PT episode number in ICU served as a protective indicator, suggesting that even if patients initially had a suboptimal response to PT, continued transfusions may lead to improved platelet increments. Similarly, higher WBC counts at ICU admission were also protective, potentially indicating a stronger immune response that mitigates damage to the transfused platelet [22].

Another important contribution of this study was the development of a nomogram to predict PT response in ICU patients, which could aid clinical decision-making. While a stratified forest plot was generated based on the multivariate GLMM analysis, the continuous variables were standardized prior to LASSO regression, limiting its direct applicability in clinical practice. In contrast, nomograms, graphical tools widely used to integrate clinical, biological, and genetic variables, offer a practical approach for estimating diagnosis and prognosis across various diseases, making them highly valuable in clinical settings [23]. To our knowledge, few studies have constructed nomograms specifically for assessing PT response in general ICU populations. In our study, the nomogram demonstrated strong predictive performance in distinguishing suboptimal from optimal PT responses. It incorporates seven routinely available clinical, imaging, and laboratory variables, which are cost-effective and accessible in most hospitals, making it highly applicable in clinical settings. This approach enables clinicians to rapidly assess patient risk and tailor treatment plans based on individual profiles during point-of-care evaluations.

Contrasted with previous studies, although no significant differences in ICU or hospital mortality or severe bleeding events (WHO grade 3–4) were observed between patients with and without suboptimal responses, our univariate GLMM analysis demonstrated suboptimal PT responses were significantly associated with higher RBC- and FFP transfusion requirements within 24 h post-PT. We attributed this to several factors: (1) Grouping: Assessing RBC/FFP requirements based solely on the presence of a single suboptimal response may not be appropriate; (2) Statistical considerations: When evaluating the impact of a single suboptimal PT on RBC/FFP requirements, random effects of the patient-level should be accounted for, rather than relying on simple chi-square or Mann–Whitney *U* test; (3) Outcome measures: It is more relevant to focus on RBC and FFP requirements within 24 h post-PT, rather than total ICU or hospital demand, given the influence of multiple factors. Additionally, we conducted a stratified 28-day survival analysis based on single and multiple transfusions, revealing different outcomes. For patients receiving a single transfusion, suboptimal transfusion response was associated with reduced survival. However, in patients receiving multiple transfusions, even those diagnosed with PTR, overall survival was unaffected. This may be due to several factors: (1) While suboptimal PT response reduces survival, continued transfusions in poor responders may still improve outcomes, as suggested by the protective effect of PT episode number in the ICU; (2) Study design: Defining the start of observation for 28-day survival in multiple transfusion patients is challenging, complicating the assessment of PTR’s impact. In this study, PTR patients were observed from the time of PTR diagnosis, while non-PTR patients were observed from the second time of transfusion. As effective transfusion often delays the second transfusion until closer to death, this group may show lower survival; (3) Suboptimal PT response may not directly reduce survival. Patients with a single suboptimal transfusion often die quickly from underlying conditions, receiving no additional transfusions and resulting in shorter survival in this group.

Our study has several strengths. First, it was conducted across six ICUs within three campuses of a single hospital, excluding patients with hematologic-related thrombocytopenia, which enhances the generalizability of our findings. Additionally, the use of the CCI as a measure of PT response was a notable strength, as it accounts for platelet dose and BSA, thereby providing a more accurate assessment of platelet increment. We established a CCI evaluation window of 10–24 h, which is more relevant to clinical practice. Furthermore, we employed GLMM to account for the random effect of the patient associated with multiple transfusions during each PT episode. Importantly, we developed a nomogram to improve the clinical applicability of our findings. This nomogram integrates seven factors, enabling individualized risk predictions for suboptimal PT response, making it a valuable tool for clinical decision-making. However, several limitations must be acknowledged. As a retrospective, single-center study, the findings may not be generalizable to other ICU settings. Although we adjusted for key clinical factors in our analysis, unmeasured variables, such as individual platelet donor characteristics, may still influence PT response. Additionally, while we used a robust multivariate model to identify predictors of suboptimal PT response, the observational nature of this study limits causal inferences. Lastly, our findings regarding the impact of suboptimal PT on patient outcomes differed from previous studies, highlighting the need for further multicenter prospective research.

## 5. Conclusions

In conclusion, our study underscored the high prevalence of suboptimal PT response in thrombocytopenic patients without primary hematologic disorders. Continued PT may enhance transfusion efficacy and improve patient outcomes, even among those classified as PTR. The development of a nomogram to predict suboptimal PT response demonstrated a good predictive performance and could provide a practical tool for enhancing PT practices in the ICU.

## Supplementary Material

Supplemental Material

## Data Availability

The data sets used and/or analyzed during the current study are available from the corresponding author on reasonable request.

## Supplementary information

Supplementary Material: Figure S1. Correlation Matrix Analysis Before and After Removal of Highly Correlated Factors. Figure S2. Variable Selection Using LASSO and Stepwise Regression. File S1. VIF values for whole variables. File S2. The fixed effects of model1 with patient-level random effect. File S3. The fixed effects of model2 with patient- and ward-level random effects. Table S1. Model Comparison Metrics for Sensitivity Analysis of Generalized Linear Mixed Models.
