# Supplementary material for: Platelet transfusion response in critically ill patients with thrombocytopenia: a retrospective study and predictive nomogram in a general ICU population
Source: Ann Med. 2025 Jul 1;57(1):2525395. doi: 10.1080/07853890.2025.2525395 (PMC12217101; doi:10.1080/07853890.2025.2525395)
Supplement: Supplemental Material [file IANN_A_2525395_SM9318.zip › suppl_data/Figure S1 and S2 caption.docx]

**Figure S1.** Correlation matrix analysis before and after removal of highly correlated factors. A. Correlation matrix of all variables; B. after removal of factors with absolute correlation coefficients > 0.7

**Figure S2.** Variable selection using LASSO and stepwise regression. A. LASSO regression results for variable selection; B. Stepwise regression results for variable selection
