# Supplementary material for: Platelet transfusion response in critically ill patients with thrombocytopenia: a retrospective study and predictive nomogram in a general ICU population
Source: Ann Med. 2025 Jul 1;57(1):2525395. doi: 10.1080/07853890.2025.2525395 (PMC12217101; doi:10.1080/07853890.2025.2525395)
Supplement: Supplemental Material [file IANN_A_2525395_SM9318.zip › suppl_data/File S1.docx]

| **File S1. variance inflation factor values for variables** |  |
| --- | --- |
|  | vif_values |
| age | 3.184758405 |
| gender | 2.273046734 |
| BSA | 2.501283925 |
| Hb on ICU admission | 2.369882602 |
| PLT on ICU admission | 2.130923137 |
| LOS of hospital before ICU | 1.566420446 |
| pa_GCS | 3.359426157 |
| pa_APACHE | 2.9375798 |
| ep_SAPS | 6.838745633 |
| WBC on ICU admission | 1.933758415 |
| on ICU admission APTT | 1.712227617 |
| on ICU admission TT | 1.960974447 |
| on ICU admission INR | 1.836426604 |
| Admission type | 2.743460568 |
| Sepsis | 2.32740478 |
| DIC | 1.730546262 |
| Trama with brian | 2.887723139 |
| Heart failure | 2.090808012 |
| Liver failure | 1.637874128 |
| Splenomegaly | 2.022715402 |
| Respiratory failure | 2.248018291 |
| Renal failure | 2.235204056 |
| Hypertension | 1.711920391 |
| Diabetes Mellitus | 1.741961591 |
| Blood infection | 1.77132253 |
| Lung infection | 1.699747391 |
| infection | 1.664178091 |
| shock | 1.897360033 |
| Solid tumor | 1.584225651 |
| ep_Mechanical Ventilation | 2.394806399 |
| ep_CRRT | 2.10637007 |
| Temperature | 1.882580016 |
| Heart rate | 1.632191721 |
| Respiration | 1.5783957 |
| Mean arterial BP | 1.671635165 |
| Urine volume | 1.783572784 |
| ep_SOFA | 8.036435587 |
| FiO_2_ | 1.988436259 |
| PaO_2_ | 2.204767391 |
| SaO_2_ | 2.011844119 |
| K^+^ | 1.605549083 |
| Ca^2+^ | 1.967310678 |
| Lactic acid | 4.026661441 |
| Potential of hydrogen | 2.323819827 |
| Anion gap | 5.860488972 |
| Glucose | 1.893418173 |
| Plasma osmolality | 2.546651931 |
| Actual bicarbonate | 3.124988962 |
| PLT_pre | 2.347749799 |
| CRP_pre | 2.368048139 |
| RDW_pre | 2.267633818 |
| MCH_pre | 1.544299175 |
| MCHC_pre | 2.040944547 |
| Hb_pre | 2.468836871 |
| Neutrophil #_pre | 5.314641136 |
| Neutrophil %_pre | 5.070726848 |
| Lymphocyte #_pre | 3.015708373 |
| Monocyte #_pre | 4.613512414 |
| Monocyte %_pre | 5.005378939 |
| Eosinophil %_pre | 2.144706069 |
| Basophil #_pre | 4.018963522 |
| Basophil %_pre | 3.10774233 |
| ALT_pre | 1.789529609 |
| ALT/AST_pre | 1.47731229 |
| Total bilirubin_pre | 2.76037821 |
| Total protein_pre | 1.862709256 |
| ALB/GLB_pre | 2.028427295 |
| Creatinine_pre | 3.050410835 |
| Urine creatinine_pre | 2.223809941 |
| Alkaline phosphatase_pre | 2.889569777 |
| GGT_pre | 1.915382459 |
| Prothrombin time _pre | 2.321543089 |
| APTT_pre | 1.982930479 |
| TT_pre | 1.709659658 |
| INR_pre | 2.726414323 |
| Fibrinogen_pre | 2.324353685 |
| D-Dimer_pre | 1.646015066 |
| NLR_pre | 2.676671548 |
| Glucocorticoids | 1.476741962 |
| Carbapenems | 1.940543338 |
| Quinolones | 1.465281407 |
| Aminoglycosides | 1.404114937 |
| Glycopeptides | 1.472122152 |
| Tetracyclines | 1.613235214 |
| Polymyxins | 1.46101398 |
| Other antibiotics | 1.396097742 |
| Antifungal agents | 1.388132358 |
| Antiviral agents | 1.211006644 |
| Procoagulant agents | 1.479096902 |
| Hemostatic agents | 1.547796919 |
| Therapeutic anticoagulants | 1.302946118 |
| bleeding grade_pre | 1.773058166 |
| Interval before testing | 1.223342257 |
| Interval after testing | 1.333053035 |
| LOS of ICU Stay at transfusion | 2.011673034 |
| RBCs with PT | 2.685027495 |
| FFP with PT | 1.611336644 |
| Cryo with PT | 1.933076524 |
| PLT rank | 2.774983253 |
| PLT duration | 1.198072239 |
| ABO type | 1.445906192 |
| death | 2.017602076 |
| bleeding | 2.218062927 |
| heal | 2.203389935 |
| *Vif* variance inflation factor, *BSA* body surface area, *Hb* hemoglobin, *ICU* intensive care unit, *PLT* platelet, *LOS* length of stay, *pa* patient’s, *GCS* Glasgow Coma Scale, *ep* episode, *WBC* white blood cell, *APTT* activated partial thromboplastin time, *TT* thrombin time, *INR* international normalized ratio, *DIC* Disseminated Intravascular Coagulation, *BP* blood pressure, *SOFA* Sequential Organ Failure Assessment, *pre* before platelet transfusion, *CRP* C-reactive protein, *RDW* red cell distribution width, *MCH* Mean Corpuscular Hemoglobin, *MCHC* mean corpuscular hemoglobin concentration, *#* count, *%* percentage, *ALT* alanine aminotransferase, *AST* aspartate aminotransferase, *ALB* albumin, *GLB* globulin, *GGT* gamma-glutamyltransferase, *NLR* neutrophil-lymphocyte ratio, *RBC* red blood cell, *PT* platelet transfusion, *FFP* fresh frozen plasma, *Cryo* cryoprecipitated antihemophilic factor | |
