# Supplementary material for: Platelet transfusion response in critically ill patients with thrombocytopenia: a retrospective study and predictive nomogram in a general ICU population
Source: Ann Med. 2025 Jul 1;57(1):2525395. doi: 10.1080/07853890.2025.2525395 (PMC12217101; doi:10.1080/07853890.2025.2525395)
Supplement: Supplemental Material [file IANN_A_2525395_SM9318.zip › suppl_data/File S2.docx]

| **File S2. The fixed effects of generalized linear mixed model with patient-level random effect.** | | | | | | | | |
| --- | --- | --- | --- | --- | --- | --- | --- | --- |
| effect | group | term | estimate | std.error | statistic | p.value | conf.low | conf.high |
| fixed |  | (Intercept) | 1.046662973 | 0.56415062 | 0.084614176 | 0.932568129 | 0.363923262 | 3.010259288 |
| fixed |  | pa_APACHE | 3.719904185 | 0.996902963 | 4.902012073 | 9.486E-07 | 2.199970797 | 6.289941288 |
| fixed |  | WBC on ICU admission | 0.690043849 | 0.112782471 | -2.269912686 | 0.023212881 | 0.500903662 | 0.950602979 |
| fixed |  | Sepsis | 4.35695197 | 1.903800008 | 3.368233554 | 0.000756515 | 1.850309708 | 10.25938003 |
| fixed |  | Liver failure | 1.095698204 | 0.384466264 | 0.260459314 | 0.794509495 | 0.550827212 | 2.179548374 |
| fixed |  | Splenomegaly | 5.711997651 | 4.731565075 | 2.10364833 | 0.035409135 | 1.126394393 | 28.96580218 |
| fixed |  | Diabetes Mellitus | 1.515457203 | 0.664694403 | 0.947806373 | 0.343228036 | 0.641502458 | 3.580049466 |
| fixed |  | ep_CRRT | 1.358265463 | 0.536863423 | 0.774708054 | 0.438512191 | 0.625952832 | 2.947322821 |
| fixed |  | Mean arterial BP | 0.871682497 | 0.136321497 | -0.87813138 | 0.379872425 | 0.641563759 | 1.184341174 |
| fixed |  | Ca^2+^ | 0.97241781 | 0.162555658 | -0.167316572 | 0.867120965 | 0.700745863 | 1.349414169 |
| fixed |  | Neutrophil %_pre | 1.262013689 | 0.215975007 | 1.359793697 | 0.173895217 | 0.902388374 | 1.764959077 |
| fixed |  | Monocyte %_pre | 0.80399148 | 0.143990701 | -1.218162648 | 0.223162171 | 0.565985974 | 1.142081836 |
| fixed |  | APTT | 0.993755971 | 0.156341771 | -0.039813383 | 0.968241907 | 0.730071219 | 1.352677525 |
| fixed |  | Polymyxins | 1.433427378 | 0.880663194 | 0.586071755 | 0.557827296 | 0.429950052 | 4.778959879 |
| fixed |  | Procoagulant agents | 1.596323031 | 0.611159294 | 1.221620754 | 0.221851076 | 0.753762496 | 3.380703119 |
| fixed |  | RBCs with PT | 1.523751923 | 0.34115356 | 1.8811682 | 0.059949042 | 0.982513076 | 2.363144045 |
| fixed |  | FFP with PT | 1.031773398 | 0.204585715 | 0.157747621 | 0.874655674 | 0.699525248 | 1.521826908 |
| fixed |  | PLT rank | 0.534879316 | 0.136066975 | -2.459682439 | 0.013906 | 0.324877919 | 0.880625818 |
| fixed |  | Interval after testing | 1.398055458 | 0.229167245 | 2.044199881 | 0.040933805 | 1.013903596 | 1.927756318 |
| fixed |  | Glucocorticoids | 0.567146602 | 0.187642805 | -1.714161534 | 0.086499063 | 0.296532142 | 1.084723114 |
| fixed |  | Hb | 1.102074164 | 0.191984815 | 0.557934777 | 0.576888923 | 0.783301237 | 1.550575188 |
| fixed |  | PLT_pre | 0.985554305 | 0.155512166 | -0.092216902 | 0.926525709 | 0.723382715 | 1.342743292 |
| fixed |  | ep_Mechanical Ventilation | 3.710818755 | 2.005703443 | 2.425992007 | 0.015266607 | 1.286453089 | 10.70398599 |
| ran_par | patientid | sd__(Intercept) | 1.532945041 |  |  |  |  |  |
| *Std* standard, *conf* confirm, *pa* patient’s, *APACHE* Acute Physiology and Chronic Health Evaluation II, *WBC* white blood cell, *ICU* intensive care unit, *ep* episode, *BP* blood pressure, *%* percentage, *pre* before platelet transfusion, *APTT* activated partial thromboplastin time, *RBC* red blood cell, *PT* platelet transfusion, *FFP* fresh frozen plasma, *PLT* platelet, *Hb* hemoglobin, *ran* random, *par* parameter, *sd* standard deviation | | | | | | | | |
