# Supplementary material for: Platelet transfusion response in critically ill patients with thrombocytopenia: a retrospective study and predictive nomogram in a general ICU population
Source: Ann Med. 2025 Jul 1;57(1):2525395. doi: 10.1080/07853890.2025.2525395 (PMC12217101; doi:10.1080/07853890.2025.2525395)
Supplement: Supplemental Material [file IANN_A_2525395_SM9318.zip › suppl_data/File S3.docx]

| **File S3. The fixed effects of generalized linear mixed model with patient- and ward-level random effect.** | | | | | | | | |
| --- | --- | --- | --- | --- | --- | --- | --- | --- |
| effect | group | term | estimate | std.error | statistic | p.value | conf.low | conf.high |
| fixed |  | (Intercept) | 1.046658234 | 0.564169624 | 0.084602543 | 0.932577377 | 0.363906923 | 3.010367183 |
| fixed |  | pa_APACHE | 3.720098803 | 0.997172419 | 4.901139019 | 9.52826E-07 | 2.199834029 | 6.290990557 |
| fixed |  | WBC on ICU admission | 0.690035565 | 0.112788907 | -2.269829343 | 0.02321794 | 0.500886564 | 0.950612602 |
| fixed |  | Sepsis | 4.357269984 | 1.904272959 | 3.367809801 | 0.000757678 | 1.850166782 | 10.26167041 |
| fixed |  | Liver failure | 1.095689817 | 0.38448096 | 0.260425552 | 0.794535535 | 0.550805617 | 2.179600459 |
| fixed |  | Splenomegaly | 5.712364556 | 4.732195554 | 2.103580702 | 0.035415039 | 1.126340541 | 28.97090857 |
| fixed |  | Diabetes Mellitus | 1.515470854 | 0.664726318 | 0.947789941 | 0.343236403 | 0.641486726 | 3.580201765 |
| fixed |  | ep_CRRT | 1.358287225 | 0.536893214 | 0.774718013 | 0.438506305 | 0.625943722 | 2.947460162 |
| fixed |  | Mean arterial BP | 0.871676847 | 0.136326582 | -0.878134374 | 0.379870801 | 0.64155099 | 1.184349393 |
| fixed |  | Ca^2+^ | 0.972419856 | 0.162561389 | -0.167298437 | 0.867135234 | 0.700739726 | 1.349431667 |
| fixed |  | Neutrophil %_pre | 1.262023492 | 0.215985885 | 1.359781158 | 0.173899187 | 0.902382489 | 1.764998008 |
| fixed |  | Monocyte %_pre | 0.803985032 | 0.143996127 | -1.218151753 | 0.22316631 | 0.565972354 | 1.142091 |
| fixed |  | APTT | 0.99375877 | 0.15634751 | -0.039794128 | 0.968257257 | 0.730065646 | 1.35269547 |
| fixed |  | Polymyxins | 1.433471288 | 0.88072359 | 0.586099374 | 0.557808737 | 0.429943577 | 4.779324651 |
| fixed |  | Procoagulant agents | 1.596346533 | 0.611193008 | 1.221609805 | 0.221855219 | 0.753750719 | 3.380855487 |
| fixed |  | RBCs with PT | 1.523767975 | 0.341170395 | 1.881142239 | 0.059952573 | 0.982506692 | 2.363209188 |
| fixed |  | FFP with PT | 1.031780788 | 0.204596637 | 0.157776448 | 0.874632958 | 0.699517692 | 1.521865147 |
| fixed |  | PLT rank | 0.53486609 | 0.136076168 | -2.459552649 | 0.013911029 | 0.324854937 | 0.880644565 |
| fixed |  | Interval after testing | 1.398077395 | 0.229187616 | 2.044145984 | 0.040939128 | 1.013895662 | 1.927831902 |
| fixed |  | Glucocorticoids | 0.567132194 | 0.187648656 | -1.714141316 | 0.086502775 | 0.296513728 | 1.084735362 |
| fixed |  | Hb | 1.102082392 | 0.191994822 | 0.557952717 | 0.576876672 | 0.783295141 | 1.550610408 |
| fixed |  | PLT_pre | 0.985550202 | 0.155516758 | -0.092240175 | 0.926507219 | 0.723372167 | 1.342751692 |
| fixed |  | ep_Mechanical Ventilation | 3.711044097 | 2.006013263 | 2.425876956 | 0.015271447 | 1.286403459 | 10.70569905 |
| ran_pars | patientid | sd__(Intercept) | 1.533071412 |  |  |  |  |  |
| ran_pars | ward | sd__(Intercept) | 0 |  |  |  |  |  |
| *Std* standard, *conf* confirm, *pa* patient’s, *APACHE* Acute Physiology and Chronic Health Evaluation II, *WBC* white blood cell, *ICU* intensive care unit, *ep* episode, *BP* blood pressure, *%* percentage, *pre* before platelet transfusion, *APTT* activated partial thromboplastin time, *RBC* red blood cell, *PT* platelet transfusion, *FFP* fresh frozen plasma, *PLT* platelet, *Hb* hemoglobin, *ran* random, *pars* parameters, *sd* standard deviation | | | | | | | | |
