# Supplementary material for: Platelet transfusion response in critically ill patients with thrombocytopenia: a retrospective study and predictive nomogram in a general ICU population
Source: Ann Med. 2025 Jul 1;57(1):2525395. doi: 10.1080/07853890.2025.2525395 (PMC12217101; doi:10.1080/07853890.2025.2525395)
Supplement: Supplemental Material [file IANN_A_2525395_SM9318.zip › suppl_data/Table S1.docx]

| Table S1. Model comparison metrics for sensitivity analysis of generalized linear mixed models. | | | | | | | |
| --- | --- | --- | --- | --- | --- | --- | --- |
|  | AIC | BIC | logLik | deviance | Chisq | Df | *P* value |
| Model 1 | 589.44 | 697.62 | -270.72 | 541.44 |  |  |  |
| Model 2 | 591.44 | 704.12 | -270.72 | 541.44 | 0.0002 | 1 | 0.989 |
| *Model 1* patient-level random effects, *Model 2* patient and ward-level random effects  *AIC* Akaike Information Criterion, *BIC* Bayesian Information Criterion, *logLik* Log-Likelihood, *Chisq* Chi-Squared Statistic, *Df* Degrees of Freedom | | | | | | | |
