# Supplementary figures and images for: Platelet transfusion response in critically ill patients with thrombocytopenia: a retrospective study and predictive nomogram in a general ICU population
Source: Ann Med. 2025 Jul 1;57(1):2525395. doi: 10.1080/07853890.2025.2525395 (PMC12217101; doi:10.1080/07853890.2025.2525395)

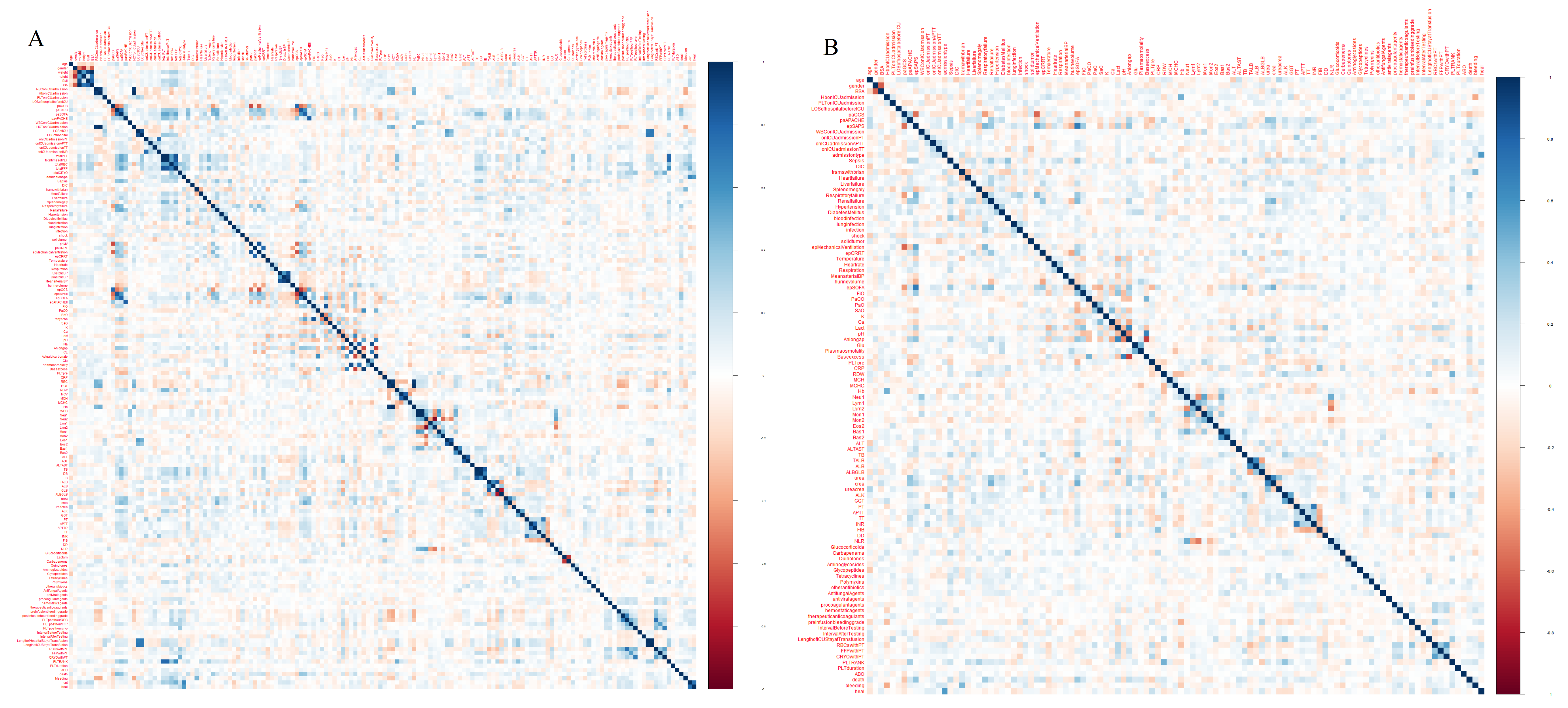

Supplement: Supplemental Material [file IANN_A_2525395_SM9318.zip › suppl_data/Figure S1.tif]

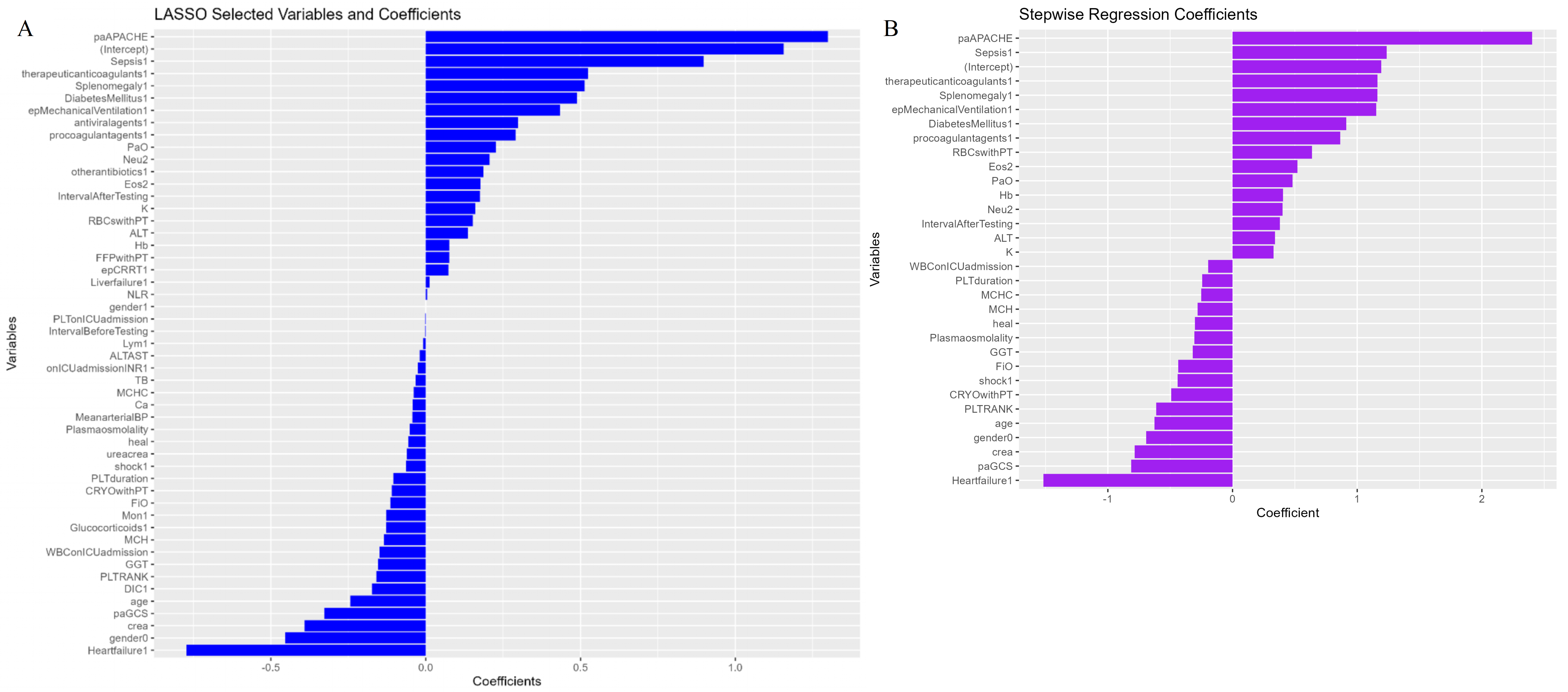

Supplement: Supplemental Material [file IANN_A_2525395_SM9318.zip › suppl_data/Figure S2.tif]
